# Adaptation of a mobile app for early anxiety and depression intervention in university students in Chile: participatory study

**DOI:** 10.1192/bji.2023.1

**Published:** 2023-05

**Authors:** Daniela Lira, Patricio Caviedes, Vania Martínez

**Affiliations:** 1 Psychologist, PhD(c), Doctoral Programme in Psychotherapy, Pontificia Universidad Católica de Chile, Santiago, Chile and Universidad de Chile, Santiago, Chile; 2 Psychologist, MSc., Research Assistant, Millennium Nucleus to Improve the Mental Health of Adolescents and Youths (Imhay), Santiago, Chile; 3 Child and Adolescent Psychiatrist, PhD, Full Professor, Centro de Medicina Reproductiva y Desarrollo Integral del Adolescente (CEMERA), Facultad de Medicina, Universidad de Chile, Santiago, Chile. Email vmartinezn@uchile.cl

**Keywords:** Depressive disorders, anxiety disorders, participatory design, mobile application, university students

## Abstract

The high prevalence of mental health problems among university students poses a challenge when developing effective interventions, with digital technologies emerging as a potential resource to address this problem. The inclusion of student input in the design and development of such interventions is critical to improving their impact. This study contributed to the initial phase of a research project that aims to adapt and evaluate the feasibility and acceptability of an early intervention for anxiety and depression based on digital technologies for university students. Three participatory workshops were conducted with 13 university students in Chile to inquire about the features and content that a mental health mobile app should include to meet their needs and preferences. The workshop transcripts were analysed using inductive thematic analysis. The results of this study highlight the value of modifications such as the personalisation of some features of the app. The students recommended incorporating topics related to university life and the possibility of contacting a mental health professional, as well as the inclusion of peer interaction or other forms of support.

Current estimates suggest that around one-third of university students worldwide have mental health problems, the most common of which are depression and anxiety.^1^ These problems have been linked to various negative consequences, such as impaired academic performance and learning,^2^ increasing risky behaviours, including problematic alcohol and drug use,^3^ suicidal ideation and related behaviours.^4^

Research has shown that mental health problems frequently occur among university students in Chile as well,^5^ and the COVID-19 pandemic led to additional difficulties and negative experiences that had an adverse effect on their mental health.^6^ Faced with this scenario, it was proposed that prevention and early intervention should be a priority public health policy, with a view to ameliorating the impact of the pandemic on the lives and well-being of students, who are so important to the future social capital of our communities.^7^ When considering how effective programmes should be developed and tailored to the needs and preferences of this population, we considered that digital technologies could provide a valuable resource, given their high acceptability, effectiveness and accessibility.^8^ However, digital interventions are seldom administered to university students, especially in Latin America, where these initiatives are in an early stage of development.^9^ In this context, we concluded that the inclusion of student input in the design and development of such interventions would be critical to increasing their impact.

## Method

**Fig. 1:**
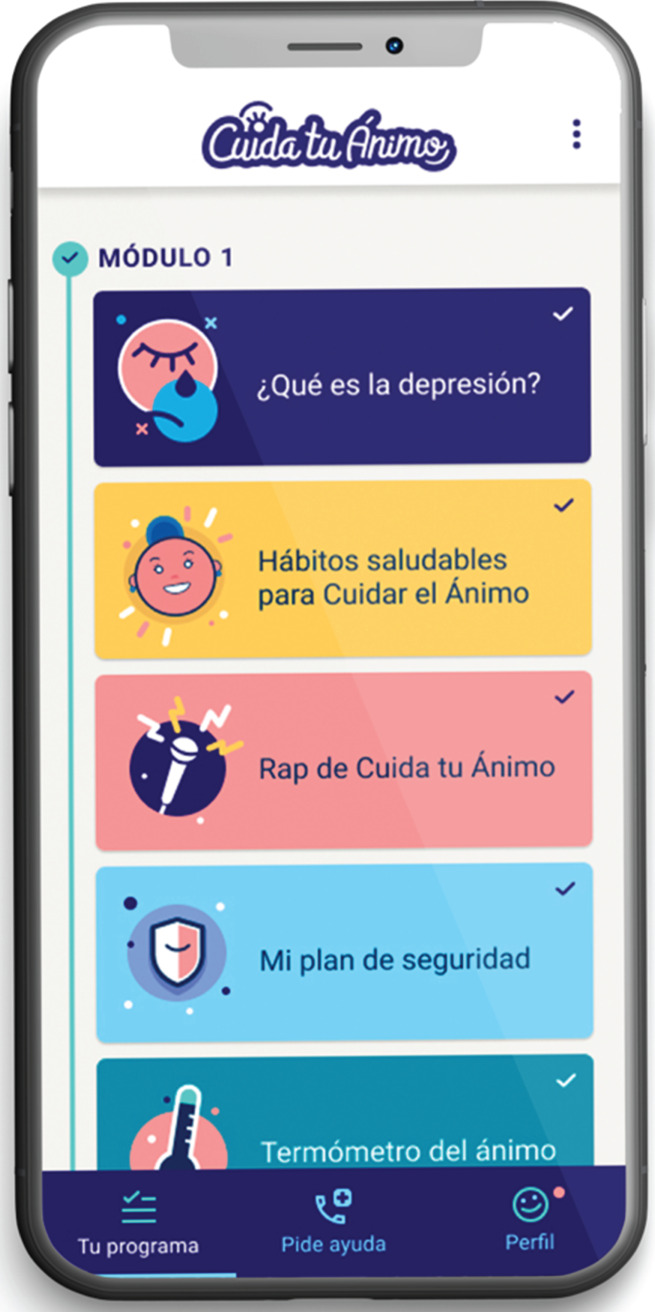
Initial version of the Cuida tu Ánimo mobile app.

**Fig. 2:**
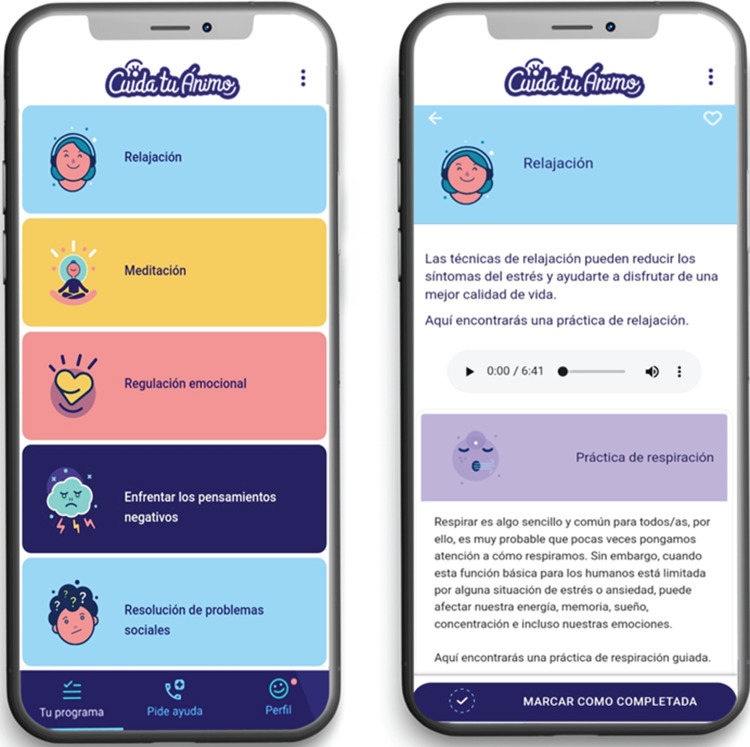
Psychoeducational components of the mobile app.

**Fig. 3:**
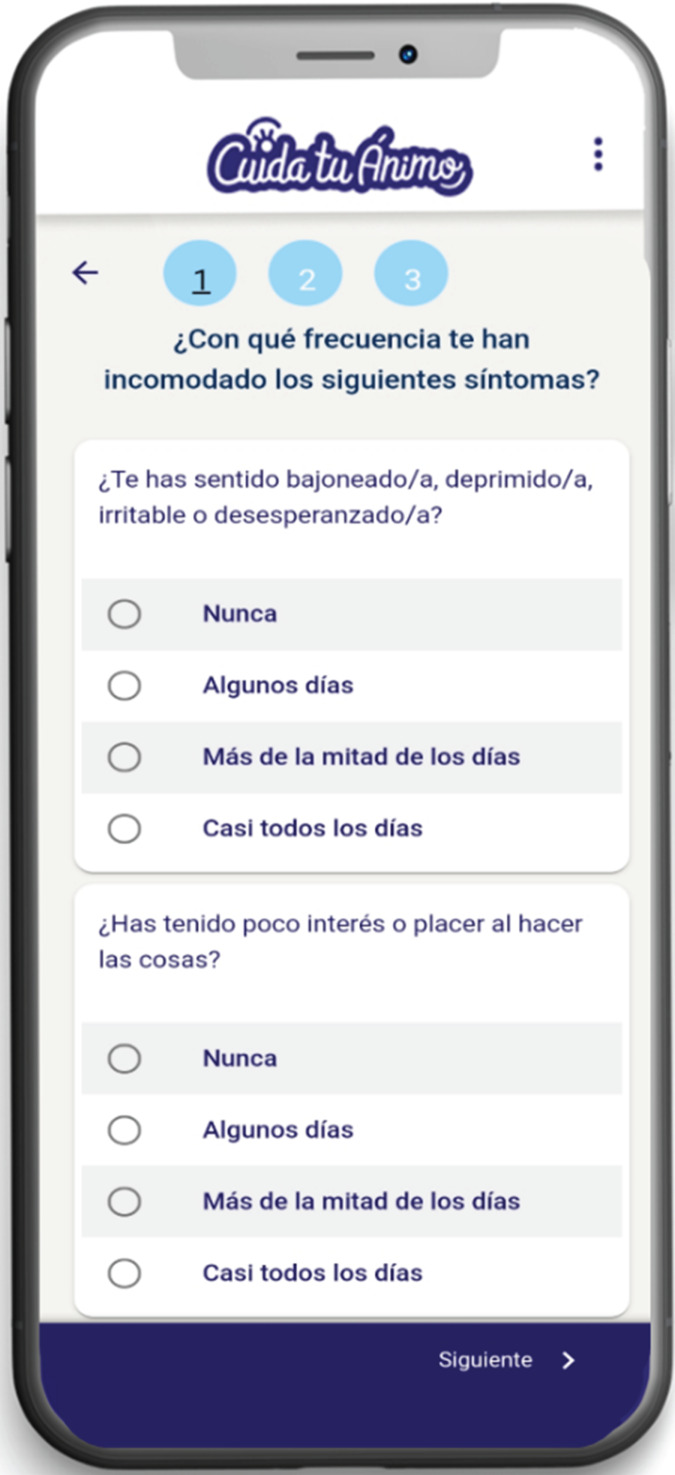
Mood monitoring of the mobile app.

This study is part of the initial phase of a research project that aims to adapt an early intervention for anxiety and depression based on digital technologies for university students and to evaluate its feasibility and acceptability. Through a participatory research design, three workshops were conducted with undergraduate students from a public university in Santiago, Chile. They were asked about the features, content and functionalities that a mobile app should contain to fit the needs and preferences of this population. The first workshop consisted of the initial adaptation of the ‘Cuida tu Ánimo’ (CTA) (Take Care of Your Mood) programme,^10^ a web-based intervention. It had previously been tested on secondary school students, and that became the initial version of the mobile app (Fig. 1). This initial version of the CTA mobile app included the following components: (a) psychoeducational information on depression, healthy lifestyle habits, emotion regulation, social support networks and cognitive–behavioural techniques (Fig. 2); (2) mood monitoring and feedback (Fig. 3); and (c) an emergency contacts section.

In response to the COVID-19 pandemic, the mobile app was adapted for use by adolescents and young people outside of school settings. Before the second and third workshops, students were asked to use a modified version of the mobile app (http://imhay.org/cuidatuanimo/), which provided the prototype for a new version that would focus on early depression and anxiety. Participants were recruited from a broader research project: the Chilean branch of the World Health Organization's WHO World Mental Health International College Student (WMH-ICS) Initiative (https://www.hcp.med.harvard.edu/wmh/college_student_survey.php). The verbal contents of the workshops were analysed using inductive thematic analysis, a strategy considered pertinent for qualitative exploratory studies, given its reflexive nature and rigour.^11^ Authors P.C. and D.L. independently reviewed and coded the transcripts using the MAXQDA software program and met periodically to compare, discuss and reach a consensus on the results of the analysis.

### Ethics

This study was reviewed and approved by the Ethics Committee of Human Subjects Research of the Faculty of Medicine, Universidad de Chile, and by the Scientific Ethics Committee of Social Sciences, Arts and Humanities of Pontificia Universidad Católica de Chile.

## Results

A total of 13 university students (9 female, 4 male) from different academic fields participated in the three workshops. Their age ranged from 19 to 32 years and they were drawn from a wide range of subject areas, including medicine, set design and law.

The students were asked about their experience with the CTA mobile app and their previous experiences with other digital mental health resources. Only one student reported having had no previous experience with digital resources. The results of the transcriptional analysis were organised into two main themes: (a) use and usability of digital resources; and (b) suggestions for improving the mobile app.

The analysis of the proceedings of the initial workshop had emphasised that students considered it important that digital resources are free, since having to pay for mobile apps discourages their use and reduces adherence. In addition, it was pointed out that the inclusion of gamification elements within digital resources promotes their use and increases adherence. Furthermore, the respondents noted that it is important for a mobile app to be endorsed by the university authorities so it could be trusted to provide reliable, evidence-based information and recommendations.

The outcome of all three workshops emphasised that students agree that digital resources have potential utility. Most students use them to monitor daily functions such as sleeping and to help them dealing with emotional discomfort, exercising, establishing healthy habits and organising their schedules. Some of them use digital resources to access information related to mental health problems. As regards the CTA mobile app, all participants felt it has potential value in identifying mental health problems, providing reliable and specific information and accessing mental health support. Most commented on its ease of use and the fact it has an aesthetically pleasing visual design. They suggested that there would be value in expanding the app's database of mental health topics to include subjects such as self-esteem and anxiety. They also suggested that it could include features that could be used to manage anxiety, such as audio resources (e.g. nature sounds).

The students highlighted the importance of incorporating topics related to university life, such as guidelines and suggestions about how to create a better balance between studies and personal life, as well as tips to improve study habits. In addition, they suggested technical improvements such as customising the notification system to make it easier to decide how often and how to use the mobile app. They also recommended that the app should provide specific information about contacting a mental health professional and should include information about peer interaction or support forums.

## Discussion

The participation of the target audience in the development and design of a mobile app makes it possible to incorporate their opinions and cater to their needs and preferences more accurately. We consider this procedure is essential to improve the usability, acceptability and engagement of mental health mobile apps, since there is otherwise a risk that they will not be acceptable, limiting their sustainability and dissemination.^12^ Thus, the results of this study highlight the importance of recommendations such as the personalisation of some features of the mobile app (e.g. notifications, frequency of use) as well as the inclusion of a wide range of topics related to mental health (e.g. self-esteem, stress) and higher education (e.g. study–life balance, study habits). In addition, the students agreed that digital interventions should include pathways that would enable them to get support from peers and mental health professionals. Forums and web chat features were thought to be useful tools that would enable students in difficulty to reach out to peers or professionals with relevant experience or expertise.

### Limitations and future research

Our findings should be interpreted with caution. First, we focused on university students. Second, we selected a relatively small sample that was limited in its social heterogeneity. Although the participating students came from different academic backgrounds, they were all enrolled in the same public university, which restricts the scope of the results. Therefore, it is necessary to increase the heterogeneity of the sample by incorporating more universities, including private universities, and diversifying the geographical location of the participating institutions. Importantly, by building on this research, future studies could shed light on a greater diversity of needs and preferences.

### Implications

The results of this study contribute to the expanding evidence for the value of developing digital technologies that can be used for prevention and early intervention for mental health problems in university students, especially in Latin America, where evidence is still scarce. We believe our findings should encourage the development of user-centred digital interventions.

## Data Availability

Data supporting the conclusions of this study are available from the corresponding author, V.M., on reasonable request.
